# Sleep Quality and Cardiopulmonary Responses During Exercise Testing: Exploring the Chronotropic and Ventilatory Response Relationship with Sleep Quality in Healthy Young Men: A Cross-Sectional Study

**DOI:** 10.3390/healthcare14010069

**Published:** 2025-12-27

**Authors:** Ahmad M. Osailan

**Affiliations:** Health and Rehabilitation Sciences Department, College of Applied Medical Sciences, Prince Sattam bin Abdulaziz University, Al-Kharj 16278, Saudi Arabia; a.osailan@psau.edu.sa

**Keywords:** sleep quality, chronotropic response, blunted autonomic response, ventilatory response, exercise testing

## Abstract

**Background:** Sleep quality is critical to health, and its disturbances may affect multiple systems, including autonomic and respiratory regulation. However, its relationship with chronotropic and ventilatory responses in healthy young men remains underexplored. Thus, the study aimed to investigate the relationship between sleep quality, as measured by the Pittsburgh Sleep Quality Index (PSQI), and chronotropic and ventilatory responses during cardiopulmonary exercise testing (CPET) in a healthy young male population and to explore group differences between good and poor sleepers. **Methods:** Thirty-three healthy men completed the PSQI and a graded CPET with breath-by-breath gas analysis. Pearson correlation was used to examine relationships between the PSQI and CPET outcomes: chronotropic response (%), tidal volume (VT), minute ventilation (VE), VO_2_, VCO_2_, expired O_2_/CO_2_, VE/VO_2_, and VE/VCO_2_. After accounting for age, height, and weight, the correlation was reassessed. Secondary analyses using a standard cut-off point compared good (PSQI < 5) vs. poor sleepers (PSQI ≥ 5) with Welch’s *t*-tests. Results: Participants were predominantly poor sleepers (84.8%; PSQI 7.3 ± 3.2). A higher PSQI correlated with lower chronotropic response (*r* = −0.35, *p* = 0.04), lower VT (*r* = −0.42, *p* = 0.02), lower expired O_2_ (*r* = −0.46, *p* = 0.01), and lower expired CO_2_ (*r* = −0.33, *p* = 0.05). Associations with VE, VO_2_, VCO_2_, VE/VO_2_, and VE/VCO_2_ were small and non-significant (*p* > 0.05). When age, height, and weight were controlled for, the attenuated chronotropic response association with the PSQI was not significant; however, the PSQI association remained significant for expired O_2_ (*r* = −0.32, *p* = 0.04), with a trend for VT. In group comparisons, chronotropic response was higher but not significant; good sleepers showed higher VT and greater expired O_2_/CO_2_ (*p* < 0.05). **Conclusions:** Poorer sleep quality was initially associated with multiple cardiopulmonary responses at peak during CPET. However, after controlling for age and anthropometry measures, only expired O_2_ remained linked. The findings suggest that routine sleep quality screening may add interpretive value to CPET by flagging individuals with reduced ventilatory depth, warranting prospective studies to test whether improving sleep quality can enhance exercise responses.

## 1. Introduction

Sleep is an essential component of health and is known to be fundamental in supporting metabolic, immune, neurocognitive, and cardiovascular homeostasis [1,2]. Because sleep is critical to many physiological functions, inadequate or disrupted sleep can disrupt multiple systems. For example, it may lead to glucose intolerance and elevated blood pressure [3]. Additionally, a blunted autonomic response has been reported among those with poor sleep quality, whereas ventilatory insufficiency has been reported in people with sleep disorders [4,5]. This sleep inadequacy may subsequently lead to an increased risk of cardiovascular morbidity and mortality [6].

There are numerous methods for non-invasively evaluating autonomic nervous system (ANS) function, including heart rate recovery (HRR), heart rate variability (HRV), and chronotropic response. Chronotropic response, which reflects the ability of the heart rate to increase in response to exercise, has been linked to adverse clinical outcomes in healthy young men and poorer outcomes in patients with coronary artery disease [4,7,8]. Sleep disturbances have been shown to alter autonomic modulation, including reduced parasympathetic reactivation (HRR) and sympathetic dominance at rest (HRV) following sleep deprivation [9,10]. Furthermore, individuals with poor sleep quality using the Pittsburgh Sleep Quality Index (PSQI) showed blunted heart rate responses during exercise [4].

Beyond autonomic modulation, sleep may affect ventilatory control and gas exchange dynamics during graded exercise [11]. The ventilatory response during exercise integrates chemoreflex sensitivity and pulmonary hemodynamics [12,13,14]. In certain clinical conditions, such as obstructive sleep apnea, impaired sleep quality can adversely affect ventilatory responses during exercise. Studies in clinical populations have shown that individuals with obstructive sleep apnea often demonstrate diminished ventilatory responses during cardiopulmonary exercise testing (CPET), which improve following appropriate treatment interventions [5]. Although the autonomic consequences of poor sleep quality have been documented, it remains underexplored whether subjective sleep quality is associated with ventilatory responses during CPET in healthy young men. A study examined the association between the PSQI and specific ventilatory responses during exercise testing in healthy adolescents and athletic adults, showing an inverse association between the VO_2_max and certain parameters of the PSQI questionnaire [15]. However, the study did not report a direct association between VO_2_max and the overall PSQI score; instead, it reported an inverse association between oxygen uptake and the PSQI at the anaerobic threshold.

Research in this area is limited; to the best of knowledge, no study has examined the relationship between overall PSQI-measured sleep quality score and multiple ventilatory responses during CPET. Thus, the current study aimed to investigate the relationship between sleep quality (PSQI) and chronotropic and multiple ventilatory responses during exercise in a healthy young adult population. The secondary aim was to explore differences in the chronotropic and ventilatory responses between good and poor sleepers.

## 2. Methods

### 2.1. Participants

The current study utilized a dataset that was previously described by Osailan et al. (2021) [16]. The methodology described in the previous study is identical to that in the current study, but it applies to other unexplored physiological outcomes. A total of 33 men without chronic health conditions were enrolled at Prince Sattam bin Abdulaziz University to participate in the study between December 2019 and December 2020. Inclusion criteria, as described by Osailan et al. (2021) [16], were individuals who were apparently healthy and over 18 years of age. Individuals were excluded if they had any cardiovascular or pulmonary diseases, conditions that might reduce lung capacity (such as a cold or flu), or sleep disorders (such as insomnia, obstructive sleep apnea, or restless legs syndrome), a history of pulmonary surgery, neurological disorders, or a recent musculoskeletal injury and any coexisting condition that would prevent safe participation in exercise testing according to the guidelines of the American College of Sports Medicine (ACSM) [17]. Additionally, individuals with medical conditions or recent surgeries that restrict their physical activity were excluded. The study was conducted in accordance with the Declaration of Helsinki and received approval from the Ethical Committee at Prince Sattam bin Abdulaziz University (RHPT/019/056). Informed consent was obtained from all participants prior to their involvement in the study.

### 2.2. Study Protocol

A total of thirty-five individuals were initially assessed for eligibility (the figure and flowchart are available from the main study by Osailan et al. (2021) [16]). Each participant attended a single session at the Exercise Research Laboratory at the College of Applied Medical Sciences, Prince Sattam bin Abdulaziz University. Upon acceptance to participate, the study protocol was provided. Participants were advised to refrain from consuming caffeinated beverages and food, as well as smoking, for at least 3 h before their test appointment, in accordance with standard exercise testing guidelines. Before undergoing exercise testing, participants completed the PSQI. After completing the questionnaires, height was measured to the nearest 0.5 cm using a stadiometer, and weight was assessed using a DETECTO weight scale (USA). Resting brachial blood pressure was measured while participants were seated and supported by an armrest using an electronic sphygmomanometer (Wollex Blood Pressure Monitor (ARM)/WXT-5902, Cigli Izmir, Turkey). Participants were then fitted with a Polar H7 (Polar Electro Oy Professorintie 5 FI-90440 Kempele, Finland) heart rate monitor to track heart rate during the exercise test and with an appropriately sized face mask to collect inspired and expired gases. Three minutes were allocated before the actual test to record resting heart rate and resting metabolic rate, as indicated by oxygen consumption while participants were seated. This was followed by administration of the graded exercise test (GXT). After completing the GXT, a recovery phase was implemented. All assessments were consistently scheduled during the daytime, specifically between 9:00 a.m. and 2:00 p.m. Two participants had their tests stopped in the early stages due to severe leg pain, without reaching voluntary exhaustion. Therefore, the final analysis included 33 participants [16].

### 2.3. Graded Exercise Test (GXT) Protocol

Cardiopulmonary exercise testing (CPET) was performed using the COSMED Quark system, which was calibrated daily to accurately measure inspired and expired gases. Participants were advised to limit their talking during the test and to respond only to questions from the examiner when prompted. The face mask was attached to the COSMED Quark CPET breath-by-breath gas analyzer. The GXT was completed on a treadmill (H/P cosmos mercury treadmill, sports & medical gmbh, Nussdoerf-Traunstien, Germany) using an individualized incremental protocol tailored to each participant’s physical capability [18]. The test began at a speed chosen by the participant (about 3.5–4 km/h) with no incline. Once initiated, three minutes were provided for familiarization and warm-up. The treadmill speed gradually increased according to each participant’s capability. By the beginning of the third minute of the familiarization period, participants were encouraged to attain their fastest comfortable walking pace (brisk walking of ~4.5–5 km/h). After the third minute, once a steady, brisk walking speed was established, the incremental phase of the test began by increasing the treadmill’s inclination. Throughout the graded exercise test, the treadmill speed remained constant, while the incline increased by 1% every minute after the third minute. Throughout the test and for at least 5 min of recovery, inspired and expired gases were measured breath-by-breath with the COSMED Quark CPET system (COSMED Quark CPET, Rome, Italy). The test was discontinued if the participant reached volitional exhaustion; if peak performance was reached (defined by a respiratory exchange ratio ≥ 1.1) and the participant was unable to continue; or if any ACSM-defined absolute or relative contraindications occurred [17]. After the test was terminated, participants sat in a chair with armrests while their heart rate and respiratory gases were monitored. Blood pressure was monitored throughout the recovery phase for at least 6 min, or until vital signs returned to baseline.

### 2.4. Outcome Measures

#### 2.4.1. Sleep Quality

Sleep quality was evaluated using the Pittsburgh Sleep Quality Index (PSQI). The questionnaire is recognized for its validity and reliability and includes 9 primary sections, each comprising 19 questions that participants complete based on their own perceptions [19]. Participants received a brief overview of the questionnaire and were offered clarification on certain items as needed. Responses were used to calculate seven key component scores: sleep quality, sleep latency, sleep duration, habitual sleep efficiency, sleep disturbances, use of sleep medication, and daytime dysfunction. Each component was scored on a 0–3 scale, yielding a total score ranging from 0 to 21 for the entire questionnaire. An overall score below 5 indicates good sleep quality, while a score of 5 or higher suggests poor sleep quality [20].

#### 2.4.2. Chronotropic Response

Chronotropic response was assessed by taking the difference between peak heart rate (HR peak) and resting heart rate (HR rest), dividing this value by the difference between the age-predicted maximum heart rate (220-age) (HR pred) and resting heart rate (HR rest), and expressing the result as a percentage [21].

$$Chronotropic\;Response\;\%=\frac{HR\;peak-HR\;rest}{HR\;pred-HR\;rest}\times 100$$

#### 2.4.3. Ventilatory Response

Ventilatory and gas exchange variables were measured during CPET using the same breath-by-breath metabolic cart and facemask system described [16]. The turbine flowmeter was calibrated with a 3 L syringe before each session; the O_2_/CO_2_ analyzers were calibrated with ambient air and a certified reference gas. Continuous breath-by-breath VO_2_, VCO_2_, VE, and VT were recorded at rest, throughout exercise, and into recovery. To reduce data variability, the final VO_2_ measurements were averaged over 30 s intervals. The highest VO_2_ recorded during the GXT was expressed as VO_2_ mL/kg/min. The averaging procedure was applied to all ventilatory response parameters, ensuring that VE, VT, VO_2_, VCO_2_, O_2_exp, and CO_2_exp values were also calculated as averages over 30 s intervals. Parameters for ventilatory response included VE, VT, VO_2_, VCO_2_, O_2_exp, CO_2_exp, VE/VCO_2_, and VE/VO_2_.

### 2.5. Sample Power Analysis

The power analysis was conducted using G*Power software (version 3.1.9.7, G*Power Team, Heinrich-Heine-University Düsseldorf, Düsseldorf, Germany). Post hoc power analysis (*t*-tests: Bivariate model; two-tailed α = 0.05; n = 33) indicated achieved power (1–β error probability) of 0.91 to detect a moderate-to-large significant effect.

### 2.6. Statistical Analysis

Data analysis was performed using IBM SPSS Statistics software (version 27, Armonk, NY, USA). The Kolmogorov–Smirnov test was used to assess the normality of the data. Variables following a normal distribution were reported as mean and standard deviation, while those not normally distributed were expressed as median and interquartile range. To address the primary aim of this study, correlations between PSQI scores and physiological outcomes were evaluated using Pearson correlation coefficients, as the PSQI, CR, and ventilatory response were normally distributed. Simple linear regression analyses were conducted to quantify the predictive contribution of the PSQI for each cardiopulmonary variable. In these models, the PSQI was entered as the independent variable and each CPET-derived physiological variable as the dependent variable. An additional bivariate correlation analysis of the same outcome measures was conducted, controlling for potential confounding variables, including age, weight, and height. These covariates were selected for their known associations with both sleep quality and cardiopulmonary responses to exercise and to estimate the association between the PSQI and physiological outcomes independent of basic demographic and anthropometric factors. To address the secondary exploration aim, and due to unequal imbalanced group sizes, between-group comparisons of CR and ventilatory response between poor sleepers (PSQI ≥ 5) and good sleepers (PSQI < 5), the comparisons were performed using Welch’s *t*-test (equal variances not assumed), which provides a more robust estimate under variance heterogeneity. The level of significance was set at *p* ≤ 0.05.

## 3. Results

The participants’ descriptive information and the main outcome measure are summarized in Table 1. The PSQI total score averaged 7.3 ± 3.2 (95% CI 6.2–8.4), indicating poor sleep quality (PSQI ≥ 5; 84.4%). Average chronotropic response was 81 ± 12% (95% CI 76.2–85.9). Generally, participants performed GXT at maximum or near-maximum effort, as indicated by an average RER of 1.14 ± 0.10 (95% CI, 1.12–1.17) at peak, along with other gas exchange parameters, including VO_2_ and VCO_2_ (Table 1).

### 3.1. Correlation Between PSQI, Chronotropic Response, and Ventilatory Response

**Figure 1:**
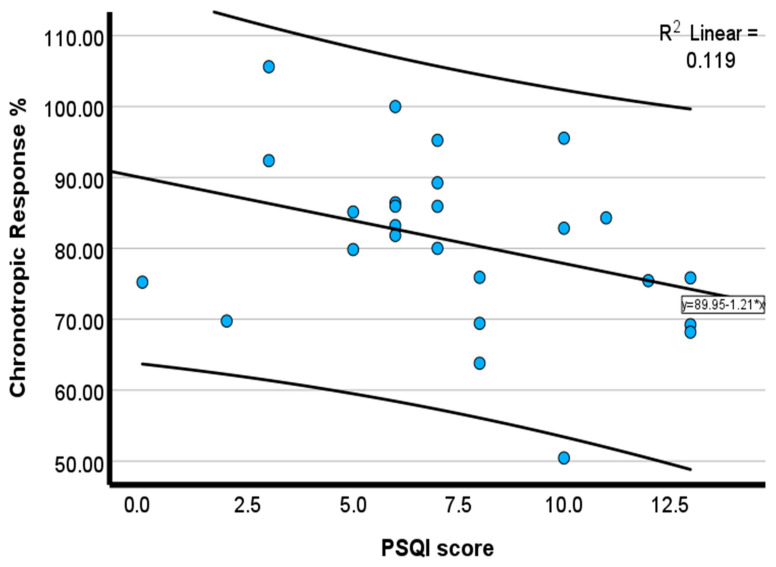
Scatterplot of the inverse relationship between the PSQI and chronotropic response. Blue circles represent individual participant data points. The solid line represents the linear regression line.

**Figure 2:**
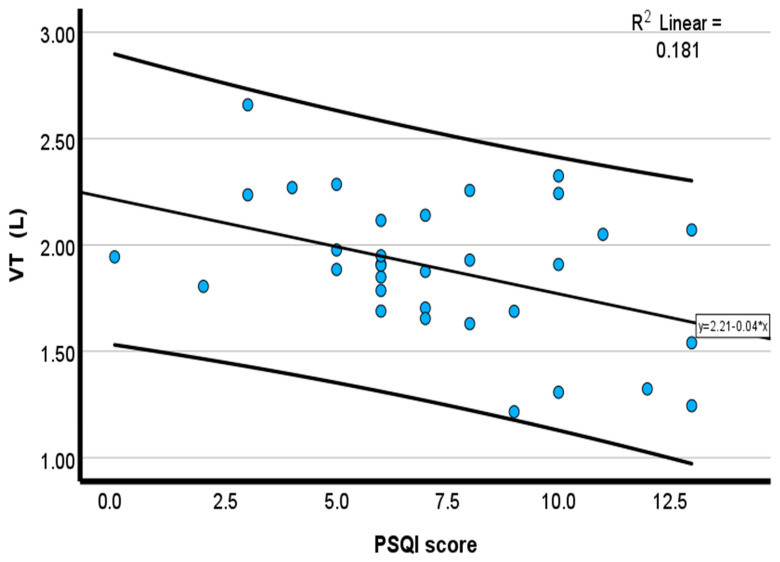
Scatterplot of the inverse relationship between the PSQI and tidal volume. Blue circles represent individual participant data points. The solid line represents the linear regression line.

**Figure 3:**
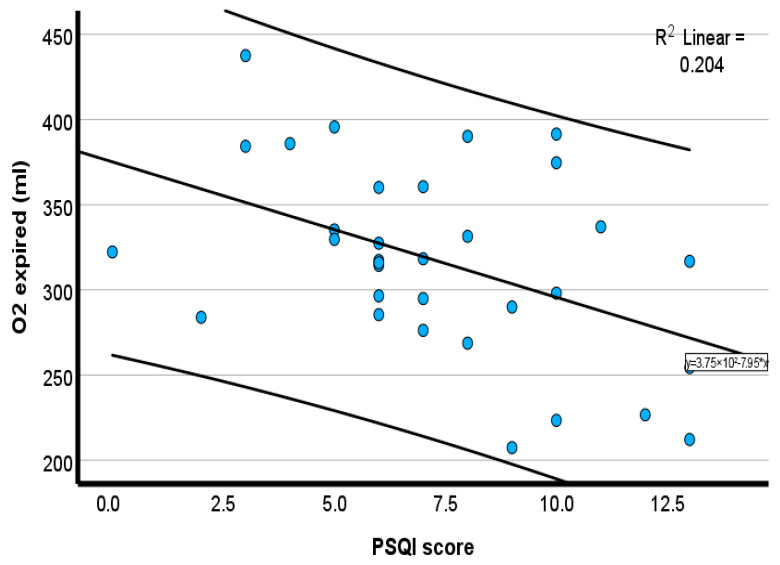
Scatterplot of the inverse relationship between the PSQI and expired oxygen. Blue circles represent individual participant data points. The solid line represents the linear regression line.

**Figure 4:**
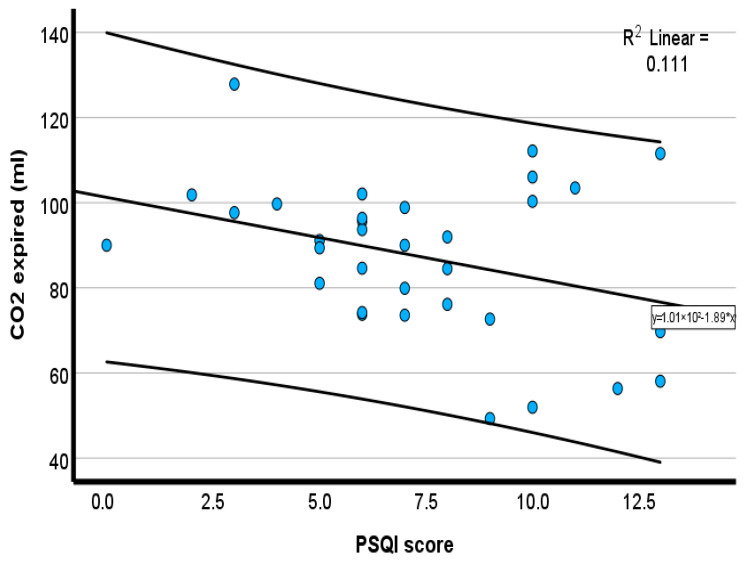
Scatterplot of the inverse relationship between the PSQI and expired carbon dioxide. Blue circles represent individual participant data points. The solid line represents the linear regression line.

Pearson correlation showed that PSQIs were moderately and inversely associated with chronotropic response (*r* = −0.35, *p* = 0.04) (Figure 1), tidal volume (*r* = −0.42, *p* = 0.02) (Figure 2), expired O_2_ (*r* = −0.46, *p* = 0.01) (Figure 3), and expired CO_2_ (*r* = −0.33, *p* = 0.05) (Figure 4) (Table 2). No significant associations were observed for minute ventilation (VE), oxygen uptake (VO_2_), carbon dioxide output (VCO_2_), or ventilatory equivalents (*p* > 0.05). To quantify the explanatory contribution of the PSQI to each CPET variable, simple linear regression models were performed, with the PSQI as the independent variable. The PSQI explained 2.9% of the variance in chronotropic response (R^2^ = 0.029, *p* = 0.40), indicating a weak and non-significant association (Table 2). In contrast, the PSQI accounted for 14.2% of the variance in tidal volume (R^2^ = 0.142, *p* = 0.03), 12.2% in expired O_2_ (R^2^ = 0.122, *p* = 0.05), and 14.3% in expired CO_2_ (R^2^ = 0.143, *p* = 0.03) (Table 2). The PSQI did not significantly explain the variance in VE, VO_2_, VCO_2_, VE/VO_2_, or VE/VCO_2_ (Table 2). The linear regression findings indicated that the PSQI accounted for a significant proportion of the variance in ventilatory responses, including VT, expired O_2_, and CO_2_. In contrast, its explanatory contribution to chronotropic response, VE, VO_2_, VCO_2_, VE/VO_2_, and VE/VCO_2_ is minimal.

The scatterplots in Figure 1, Figure 2, Figure 3 and Figure 4 collectively demonstrate a modest inverse association between PSQI scores and cardiopulmonary responses during peak exercise. Chronotropic response (Figure 1; R^2^ = 0.119) and tidal volume (Figure 2; R^2^ = 0.181) both declined with an increasing PSQI, indicating reduced chronotropic response and tidal volume among individuals with high PSQI scores (poorer sleep quality). Similar negative trends were observed for expired O_2_ (Figure 3; R^2^ = 0.204) and expired CO_2_ (Figure 4; R^2^ = 0.106), with higher PSQI scores associated with lower expired O_2_ and CO_2_. These findings suggest that poorer sleep quality may be associated with reduced chronotropic and ventilatory responses during exercise testing. After adjusting for age, height, and weight, the association with chronotropic response (r = −0.22, *p* = 0.16) was small and not significant. In contrast, higher PSQI scores were significantly associated with lower expired O_2_ at peak exercise (*r* = −0.32, *p* = 0.04), and a similar trend was seen for tidal volume (r = −0.258, *p* = 0.08), but it was not significant (data not reported).

### 3.2. Comparison Between Good and Poor Sleepers

These group comparisons were performed using Welch’s *t*-tests, considering the unequal sample sizes between good sleepers (n = 5) and poor sleepers (n = 28). Given the small number of good sleepers, these analyses should be interpreted as exploratory and may lack sufficient statistical power. No significant differences were found for chronotropic response, VE, VO_2_, VCO_2_, VE/VO_2_, or VE/VCO_2_ (*p*> 0.05). However, poor sleepers showed lower expired CO_2_ volume compared with good sleepers (Welch *p* = 0.048), with a large effect size (Hedges g = 1.07). VT and expired O_2_ volume also demonstrated large effect sizes (g = 1.04 and 0.93, respectively) (Table 3). Given the small size of the good sleeper group (n = 5), these comparisons should be interpreted as exploratory. Overall, these exploratory findings suggest that ventilatory gas exchange variables may be more sensitive to variations in sleep quality than the chronotropic response in this population 

## 4. Discussion

The current study examined the relationship between subjective sleep quality (measured by the PSQI) and the chronotropic and ventilatory responses during CPET in a healthy young male population. Additionally, for exploratory purposes, the study compared chronotropic and ventilatory responses between good and poor sleepers using established PSQI cut-off points. Initial correlation analysis without adjustment showed that poorer subjective sleep quality (higher PSQI scores) was associated with reduced chronotropic and ventilatory responses during exercise, including lower tidal volume and lower expired O_2_ and CO_2_. Consistent with these associations, simple linear regression analyses showed that the PSQI explained only a small proportion of the variance in chronotropic response but a larger proportion in ventilatory parameters, including tidal volume and expired O_2_ and CO_2_. However, after adjusting for age, height, and weight, the association with chronotropic response was directionally negative but not statistically significant. Poorer sleep quality remained significantly associated with lower expired O_2_, with a concordant trend for lower tidal volume. In group comparisons, good sleepers exhibited a non-significantly higher chronotropic response than poor sleepers. Furthermore, good sleepers achieved greater tidal volume and higher expired O_2_ and CO_2_ at peak exercise, while minute ventilation, oxygen uptake, carbon dioxide output, and ventilatory equivalents remained comparable. Overall, these findings may suggest that better sleep quality is associated with a favorable cardiopulmonary physiological response during exercise testing.

The initial inverse association (unadjusted for age and anthropometric measures) between the PSQI and chronotropic response is consistent with studies showing that poor sleep quality is associated with blunted autonomic reactivity to exercise. In a previous study using the PSQI to assess sleep quality and cardiovascular responses during a treadmill stress test in 113 apparently healthy young men, the PSQI was inversely associated with the rise in HR during exercise (also known as chronotropic incompetence) and with heart rate recovery (HRR) post-test. Additionally, poor sleepers had a smaller HR rise during exercise than good sleepers [4]. In the context of attenuated autonomic modulation, impaired post-exercise HRR, reflecting depressed parasympathetic reactivation, was reported in an experimental sleep deprivation study [9]. Furthermore, a recent systematic review and meta-analysis reported that sleep loss influenced heart rate variability (HRV) toward sympathetic predominance with vagal withdrawal (lower RMSSD, higher LF or LF/HF ratio) [22]. Studies examining the association between the PSQI and chronotropic response are scarce, as most have focused on HRV and HRR. Therefore, the current study may contribute to the existing evidence exploring the association between sleep quality and chronotropic response. However, interpretation of the results of the current study should be made with caution, as in the present cohort, the inverse association between the PSQI and attenuated chronotropic response was weak and not statistically significant after adjusting for age, height, and weight. This suggests that, in healthy young men, age and anthropometric variables may influence this relationship.

The loss of association between the PSQI and the chronotropic response after adjusting for age, height, and weight suggests that sleep quality alone may not be strongly associated with heart rate regulation during exercise testing in this cohort of healthy young men. This interpretation is consistent with the linear regression findings, in which the PSQI accounted for 2.9% of the variance in chronotropic response, indicating less explanatory power for heart rate dynamics relative to other ventilatory measures. Chronotropic response is known to worsen with increasing age [22] and higher body weight and body mass [23,24], likely due to alterations in β-adrenergic pathways. Thus, the initial observation of an inverse association between the PSQI and chronotropic response may reflect shared physiological pathways with age and anthropometric parameters. After accounting for these variables, the association between the PSQI and chronotropic response became minimal. Additionally, given the modest sample size and narrow age range, these adjusted analyses may be underpowered to detect minor independent effects. In contrast, the sustained associations between the PSQI and one measure of ventilatory response (expired O2) after the same adjustments may indicate that sleep quality was correlated with breathing patterns rather than the dynamic increase in heart rate during exercise testing. Therefore, a larger, heterogeneous sample is needed to clarify whether sleep quality is associated with chronotropic responses in healthy young men.

Several potential mechanisms may explain the initial inverse relationship between sleep quality and the chronotropic response. Poor sleep quality habits, such as repeated awakenings or microarousals, have been linked to increased sympathetic activity, decreased parasympathetic (vagal) tone, and alterations in vascular control [23]. Because the chronotropic response depends on β-adrenergic-mediated sympathetic drive and vagal withdrawal, disturbances in these processes may reduce the dynamic HR reserve available for HR increase during exercise [24,25]. Additionally, chronic sleep loss may be associated with changes in arterial baroreflex sensitivity, which could contribute to a less pronounced reflex tachycardia as arterial pressure increases during exercise and may reduce heart rate responsiveness when exercise catecholamines are released [22]. These mechanisms remain speculative and should not be interpreted as established pathways, particularly given the analyses of confounding variables in the current study—such as age, height, and weight—which indicated that the association between sleep quality and chronotropic response was modest in healthy young men. This speculation needs to be explored in larger prospective studies to explore this association further.

To the best of our knowledge, this is the first study to examine the relationship between the PSQI and multiple parameters of ventilatory response during CPET in healthy young men, underscoring the novelty of this study. Previous studies investigating the influence of subjective sleep quality on cardiopulmonary response in healthy individuals have typically reported only a limited set of ventilatory responses, most often minute ventilation or VO_2_max. Experimental studies in healthy people have shown that sleep loss can influence ventilatory response; for example, a single night of sleep deprivation reduced minute ventilation during exercise in trained athletes [26]. Similarly, studies examining subjective sleep quality have reported that individuals with better PSQI scores achieve higher maximal power output and greater VO_2_max during incremental exercise testing [27], and modest associations between the PSQI and VO_2_ or VO_2_max have also been observed in adolescent and adult athlete samples [15]. In contrast, the current study found no association between the PSQI and VO_2_, which may be due to variation in sample size and population characteristics.

Several studies in the literature have examined sleep quality in clinical cohorts and have primarily used one or two ventilatory response indices (especially VE/VCO_2_ slope). For example, a previous study in elderly patients with heart failure evaluated the PSQI and VE/VCO_2_ slope and reported concurrent improvements in sleep quality and ventilatory efficiency after low-intensity aerobic exercise, suggesting that better sleep quality may accompany more favorable ventilatory responses to exertion [28]. Similar results were reported among patients with pulmonary artery hypertension, in which 12 weeks of aerobic training improved sleep quality and increased VO_2_max [29]. A similar positive impact of exercise training has been reported in patients with chronic obstructive pulmonary disease and patients with depressive disorders [30,31]. Another interventional study investigated the effect of positive airway pressure on the VE/VCO_2_ slope in patients with obstructive sleep apnea and reported a greater reduction in the VE/VCO_2_ slope than in those not using positive airway pressure [32]. This may support a potential association between improved sleep quality and enhanced ventilatory response during exercise. Collectively, previous studies in clinical populations have indicated that a less efficient ventilatory response was observed among those with poor sleep quality. The current study’s findings contribute to this body of literature by suggesting that, among healthy young men, higher PSQI scores may be associated with a shallower ventilatory response at peak exercise, as indicated by lower tidal volume and expired gas volume.

Notably, in the current study, no association was found between the PSQI and commonly used ventilatory response indices (VE/VO_2_, VE/VCO_2_) in clinical populations. It can be speculated that this is because these indices, which rely on the peak ratio, are less sensitive to changes (more useful in a clinical cohort) than other ventilatory indices that rely on response changes (VT and expired O_2_ and CO_2_).

A plausible mechanism for the association between a poor PSQI score and ventilatory responses, including tidal volume and expired gases, may involve attenuated autonomic activation and potential changes in chemoreflex or respiratory drive that could lead to shallower breaths during exercise. Therefore, poor sleep may be linked to altered chemoreflex control, which could contribute to shallower breathing and lower expired O_2_ during exertion [26,33]. However, this interpretation remains speculative and should be considered a hypothesis for future investigation rather than a definitive explanation.

It is noteworthy that, although the group comparisons were statistically underpowered, the effect size estimates provide additional context for the direction and magnitude of the observed differences. Large effect sizes were observed for tidal volume and expired CO_2_ (g = 1.10–1.11), and a similarly large effect was noted for expired O_2_ (g = 1.00), despite the latter not reaching statistical significance. VO_2_ also demonstrated a moderate-to-large effect (g = 0.69), suggesting that good sleepers tended to achieve greater ventilatory gas exchange efficiency during peak exercise. In contrast, chronotropic response, VE, and VCO_2_ showed small-to-moderate effects, indicating weaker distinctions between sleep groups. Given the small and uneven distribution of good sleepers (n = 5) versus poor sleepers (n = 28) in our data, these between-group comparisons should be interpreted as exploratory and preliminary, supporting the possibility of meaningful physiological differences associated with poor sleep quality. This requires examination in a larger, more evenly distributed sample to verify these differences and assess their clinical relevance.

Given the observed associations between poorer subjective sleep quality and attenuated cardiopulmonary responses during exercise, this study introduces some clinical implications. Routine screening of sleep quality using the PSQI during CPET may help flag individuals with reduced ventilatory depth. CPET interpretation should consider VT and expired O_2_/CO_2_ when the PSQI is elevated. Clinically, this may prompt sleep education counselling or behavioral therapies to improve sleep quality, and, where indicated, screening for sleep disorders, such as obstructive sleep apnea. This could help identify individuals who might benefit from sleep assessment or education, but whether improving sleep leads to better exercise outcomes requires longitudinal confirmation. In the context of rehabilitation, breathing exercises may help as an intervention to improve ventilation depth and potentially sleep quality. However, whether sleep-focused or ventilatory response interventions causally improve CPET responses requires prospective longitudinal studies to investigate their long-term effects.

This study has several limitations. The cross-sectional design does not allow for causal inference regarding the relationship between sleep quality and cardiopulmonary response. One major limitation of the current study is the marked sample imbalance (good sleepers, n = 5, vs. poor sleepers, n = 28), which reduces statistical power for group comparisons; therefore, these findings should be interpreted with caution and considered exploratory. The sample comprised mostly young men, which limits generalizability to women and other age groups. Sleep quality was measured subjectively using the PSQI, although it was validated and commonly used; other objective measures (e.g., actigraphy/polysomnography) would have added greater precision and the ability to detect specific sleep disorders. Therefore, future work should use larger, balanced, more representative samples and include objective sleep assessments. Additionally, future longitudinal interventional studies should investigate whether sleep-focused or ventilatory management improves cardiopulmonary responses during CPET.

## 5. Conclusions

In conclusion, the current study demonstrated that poorer subjective sleep quality, as assessed by the PSQI, was associated with attenuated cardiopulmonary responses during exercise testing in healthy young men, which was particularly reflected in reduced ventilatory response (lower tidal volume and expired O_2_). The inverse relationship between sleep quality and cardiopulmonary responses highlights a potential pathway through which poor sleep quality may compromise physiological responses during CPET, even in healthy individuals. These findings provide novel evidence that poor sleep quality is associated with ventilatory responses during CPET in a non-clinical cohort.

## Figures and Tables

**Table 1:** Participant characteristics and cardiopulmonary exercise responses.

Variable	Value	95% Confidence Interval for Mean (Lower Bound–Upper Bound)
Age (years)	23 (22–24)	
Weight (kg)	81.9 ± 17.9	(75.6–88.3)
Height (m)	1.72 ± 0.07	(1.69–1.74)
PSQI total score	7.3 ± 3.2	(6.2–8.4)
Good sleep quality (n, %)	5, 15.2%	
Poor sleep quality (n, %)	28, 84.8%	
Resting HR (bpm)	77 (70–80)	
Resting SBP (mmHg)	127 ± 9.9	(123.1–130.5)
Resting DBP (mmHg)	83 (77–89)	
HR maximum (bpm)	169 ± 13.6	(164–174)
Chronotropic response %	81 ± 12	(76.2–85.9)
RF b/min	39.4 ± 7.1	(36.8–41.9)
VT (L)	1.9 ± 0.3	(1.8–2)
VE L/min	74 ± 18.1	(67.6–80.4)
VO_2_ mL/min	2280.9 ± 488.3	(2107.8–2454.1)
VCO_2_ mL/min	2609.3 ± 566.7	(2408.4–2810.3)
O_2_ expired mL	317.1 ± 56.2	(297.1–337)
CO_2_ expired mL	87.4 ± 18.1	(81–93.9)
VE/VO_2_	33.6 ± 4.4	(31–34.2)
VE/VCO_2_	28.4 ± 2.7	(27.4–29.3)
RER	1.14 ± 0.1	(1.12–1.17)

Values are presented as mean and standard deviation or median (25th to 75th percentile) as appropriate. PSQI; Pittsburgh Sleep Quality Index, HR; heart rate, bpm; beat per minute; SBP; systolic blood pressure, DBP; diastolic blood pressure, RF; respiratory frequency, VT; tidal volume, VE; minute ventilation, VO_2_; volume of oxygen, VCO_2_; volume of carbon dioxide, RER; respiratory exchange ratio.

**Table 2:** Correlation analysis between PSQI chronotropic response and ventilatory response.

Variable	Pearson Correlation r	*p* Value	95% Confidence Intervals	Linear Regression	
				R^2^	*p* Value
Chronotropic response	−0.35	0.04	(−1.000, −0.010)	0.029	0.4
VT (L)	−0.42	0.02	(−1.000, 0.101)	0.142	0.03
VE L/min	−0.19	0.17	(−1.000, 0.150)	0.013	0.53
VO_2_ mL/min	−0.11	0.29	(−1.000, 0.230)	0.062	0.16
VCO_2_ mL/min	−0.19	0.17	(−1.000, 0.150)	0.037	0.28
Expired O_2_ mL	−0.46	0.01	(−1.000, −0.140)	0.122	0.05
Expired CO_2_ mL	−0.33	0.05	(−1.000, 0.011)	0.143	0.03
VE/VO_2_	−0.10	0.31	(−1.000, 0.239)	0.042	0.26
VE/VCO_2_	0.002	0.47	(−0.317, 1.000)	0.028	0.36

VT, tidal volume; VE, minute ventilation; VO_2_, oxygen uptake; VCO_2_, carbon dioxide output; VE/VO_2_ and VE/VCO_2_, ventilatory equivalents.

**Table 3:** Group comparisons of cardiopulmonary response (good sleepers vs. poor sleepers).

Variable	*t*	Good Sleepers Mean ± SD	Poor Sleepers Mean ± SD	Mean Difference (Good–Poor)	95% Confidence Intervals	*p*	Hedges’ g
Chronotropic response (%)	0.65	85.7 ± 16.4	80.2 ± 11.3	5.57	(−19.4, 30.5)	0.5	0.46
VT (L)	2.27	2.2 ± 0.3	1.84 ± 0.3	0.35	(−0.06, 0.75)	0.04	1.10
VE (L·min^−1^)	0.67	78.8 ± 17.3	73.2 ± 18.5	5.57	(−15.35, 26.49)	0.54	0.30
VO_2_ (ml·min^−1^)	1.88	2563.9 ± 335.5	2230.4 ± 498.58	333.49	(−78.8, 745.7)	0.10	0.69
VCO_2_ (ml·min^−1^)	1.30	2863 ± 451.8	2564 ± 579.9	298.99	(−260.5, 859.5)	0.24	0.53
Expired O_2_ (ml)	1.88	362.8 ± 60.04	308.9 ± 52.5	53.85	(−19.2, 126.9)	0.09	1.00
Expired CO_2_ (ml)	2.61	103.4 ± 14.4	84.6 ± 17.4	18.83	(1.36, 36.30)	0.02	1.11
VE/VO_2_	−1.24	30.5 ± 4.02	32.9 ± 4.1	−2.46	(−6.78, 1.87)	0.23	−0.56
VE/VCO_2_	−0.91	27.4 ± 2.8	28.6 ± 2.7	−1.23	(−3.90, 1.44)	0.40	−0.45

Values are presented as means ± standard deviations. Welch’s *t*-test (equal variances not assumed) is reported. Effect size reported as Hedges’ g. Significant results are reported (*p* < 0.05).

## Data Availability

The raw data supporting the conclusions of this article will be made available by the authors upon request.
